# Supplementary material for: Training for endovascular therapy of acute arterial disease and procedure-related complication: An extracorporeally-perfused human cadaver model study
Source: PLoS One. 2024 Feb 8;19(2):e0297800. doi: 10.1371/journal.pone.0297800 (PMC10852297; doi:10.1371/journal.pone.0297800)
Supplement: Attachment — Submitted filename: Response to Reviewers.docx [file pone.0297800.s001.docx]

We would like to thank the reviewers for their efforts and suggestions.

Below you will find the point-by-point responses to your comments.

**Reviewer #1:**

1. The title seems in need of correction.

Since “pathology” has a strong pathological feel, it would be better to change it to “acute arterial disease.” It would be good to change “complication” to “procedure-related complication” or periprocedural complication.

Since the title is too long, I think it could be “Training for endovascular therapy of acute arterial disease and procedure-related complication: An extracorporeally-perfused human cadaval model study.”

The title was changed according to the recommendation.

2. Line 4; Please also edit “pathologies and complications” in the 4th line to fit the title.

The line has been adjusted accordingly.

3. Line 5; I think “arterial” would be better instead of “vascular.”

The words were exchanged.

4. Line 8; I think "arterial embolization" is a better word for this topic than "vessel-occluding".

Please make the same corrections to lines 19, 34, 101, 105, 110, 127, 228 and the table.

The naming has been adjusted and subsequently amended.

5. Line 10; I think it would be better to use “endovascular recanalization” rather than “vessel-opening”.

Regarding this, please make the same corrections to lines 19, 34, 101, 102, 183, 228 and the table.

The naming has been adjusted and subsequently amended.

6. Line 13; Please also add “endovascular dissection repair system” before “covered stent.”

The phrase has been added.

7. Line 19, 230; “Haptics” refers to a technology that generates vibration, force, or shock in various digital devices to give users a sense of touch. Since it does not fit with the main text, it would be better to delete it or edit it with other words.

The term should refer to the sensory experience of the devices while usage, e.g. slipping of the catheter into a side branch. As this is a relevant advantage over other models, we have decided to retain the text passage and replace the word "haptic" with "tactile".

8. Line 22; Rather than simply writing “intervention,” it would be better to change it to “endovascular procedure” or “endovascular intervention.”

The wording has been changed accordingly.

9. Line 22; Please change it to “thrombectomies” rather than “thrombosis-inducing procedures.”

The wording has been changed accordingly.

10. Line 35; I think it would be better to express it as “acute limb ischemia” rather than “acute ischemia of the vascular periphery.”

The wording has been changed accordingly.

11. Line 46-47; Rather than “emergency intervention,” “emergent endovascular treatments” seems to be a more appropriate term for the paper.

The wording has been changed accordingly.

12. Line 53; I think “Mock circulatory loops” is a more appropriate term for the paper than “Simplified fluid mock models.”

The term has been changed accordingly.

13. Line 79; Please write the product name and manufacturer of the sheath used.

The product name and manufacturer have been added.

14. Line 152; Please change “liquid embolic” to “liquid embolic material” or “liquid embolic agent.”

The wording has been changed accordingly.

15. Line 186; Since this is an American paper, please change “heamostatic” to “hemostatic.”

The spelling has been corrected.

16. Figure 8; To match the order of product description with the main text, the order of Figures B/C and Figures D/E must be changed.

The text passage has been adjusted and is now in the same order as the figure.

17. Line 229; It would be good to change “complication” to “procedure-related complication” or periprocedural complication.

The wording has been changed accordingly.

18. Line 252; Please change “intervention” to “procedure” or “treatment.”

The wording has been changed accordingly.

19. Line 280; Please change “liquid tissue adhesive” to “liquid embolic agent.”

The wording has been changed accordingly.

20. Line 295; Rather than simply writing “intervention,” it would be better to change it to “endovascular procedure” or “endovascular intervention.”

The wording has been changed accordingly.

21. Were the products used in the procedure purchased with research funds or donated by the manufacturer?

The materials and devices used had passed their expiration date and were no longer suitable for patient care for hygienic reasons but were kept for demonstration and teaching purposes and used in this task for this study.

**Reviewer #2:**

Nice paper and idea is quite interesting.

The cadaver training is well established in open surgery particularly in vessel exposure and dissection.

There is no much mentioned in literature about endovascular intervention using cadavers.

I still believe that it will not be the same as interventions in real life as different settings and different pathologies particularly hard calcification angulated vessels. However, doing this training can get the operators to become familiar with different techniques and instruments.

Another limitation is the availability of such suitable cadavers that the author should address that and how to overcome this problem.

Thank you for the positive criticism. The fact of limited availability of body donors has been further elaboration in the “Limitation” section.
